# Event-Based Camera Modeling for Atmospheric Turbulence Prediction

**DOI:** 10.3390/s25237276

**Published:** 2025-11-28

**Authors:** Dor Mizrahi, Daniel Brisk, Yogev Mordechai, Or Maor

**Affiliations:** 1 Applied Physics Division, Soreq Nuclear Research Center, Yavne 81800, Israel; 2 Department of Industrial Engineering and Management, Ariel University, Ariel 40700, Israel

**Keywords:** event-based vision sensor, atmospheric optical turbulence, path-integrated $C_{n}^{2}$ estimation, neuromorphic camera, machine-learning regression

## Abstract

Atmospheric turbulence degrades long-range imaging and free-space optical performance, yet conventional measurement systems such as large-aperture scintillometers require active transmitters, precise alignment, and dedicated deployment. This study investigates whether a passive neuromorphic event camera can provide reliable estimates of the refractive-index structure parameter $C_{n}^{2}$ along a 300 m horizontal path. We conducted a week-long field experiment using a Prophesee EVK-4 HD event camera (Prophesee, Paris, France), a Basler acA2040-120um HD CMOS video camera (Basler AG, Ahrensburg, Germany), and a Scintec BLS900 scintillometer (Scintec AG, Rottenburg, Germany) as ground truth. A compact set of 19 statistical event-stream features was extracted over multiple integration times (2–50 s), and machine learning regression models were trained to predict the corresponding scintillometer-measured turbulence. Across the full turbulence range $10^{-14}–10^{-12}\;m^{-2/3}$, the best-performing model (XGBoost) achieved a Pearson correlation of 0.93 and a mean absolute relative error of 35%, with longer integration times and higher-contrast regions yielding improved accuracy. The results also quantify, for the first time in field conditions, how integration time, target contrast, and feature stability influence event-based turbulence estimation. These findings demonstrate that passive event-driven sensing can approximate scintillometer-level turbulence measurements without active illumination, enabling compact, low-power alternatives for real-time atmospheric monitoring.

## 1. Introduction

Atmospheric turbulence—random, scale-dependent fluctuations of the air’s refractive index driven by temperature and wind gradients—imposes time-varying phase errors that blur imagery, distort geometry and reduce contrast in long-range optical systems [1,2]. For remote sensing, free-space optical communications, border surveillance and astronomical imaging, knowing the path-integrated turbulence strength, expressed by the refractive-index structure parameter $C_{n}^{2}$, is vital for performance prediction and adaptive correction [3]. Large-aperture scintillometers such as the Scintec BLS900 remain the de facto standard for measuring $C_{n}^{2}$ in the field, but they require a precisely aligned transmitter–receiver pair, dedicated power and clear lines of sight, which limit practical deployment [4].

To reduce hardware complexity, researchers have explored passive video-based estimation: by analyzing apparent turbulence-induced image motion (centroid wander), blur kernels or angle-of-arrival fluctuations in frame sequences, algorithms can correlate visual distortions with $C_{n}^{2}$ [5,6]. Yet conventional CMOS (complementary metal–oxide–semiconductor) or CCD (charge-coupled device) sensors sample the scene at fixed, relatively low frame-rates (≈30–180 frames per second (fps)) and suffer from motion blur and ≈60 dB dynamic range, so the subtle, sub-millisecond scintillation events that encode turbulence are often missed or buried in noise [7].

Event-driven vision sensors (neuromorphic or event cameras) circumvent these limitations by reporting asynchronous logarithmic brightness changes at each pixel with microsecond latency and more than 120 dB dynamic range [8,9]. Because events are generated only when and where intensity changes, the data stream is sparse, virtually free of motion blur and naturally emphasizes the fast, low-contrast fluctuations characteristic of optical turbulence. Despite this apparent fit, field studies that exploit event cameras for quantitative turbulence sensing remain limited: early laboratory work visualized hot-plate plumes [10], while Polnau and Vorontsov demonstrated feasibility over a 7 km path but relied on hand-crafted event statistics and a single acquisition configuration [11]. To the best of our knowledge based on the literature surveyed, a systematic outdoor assessment of how acquisition parameters—particularly integration time and target contrast—affect event-based turbulence estimation accuracy has not yet been reported; here we explicitly quantify these effects across multiple turbulence ranges.

The present study fills this gap by conducting a week-long campaign (27 March–3 April 2025) at Yavne, Israel, using a Prophesee Metavision Evaluation Kit 4 (EVK-4) event camera (Sony IMX636A, 1280 × 720 px, <100 µs latency, >120 dB dynamic range) co-sited with a BLS900 scintillometer on a 300 m horizontal path run near the ground at sea level. Five event-integration windows (2, 5, 10, 30 and 50 s) and three target-board contrast levels (low, medium, high) were tested to explore the trade-off between temporal resolution, event-rate sparsity and predictive reliability. Rather than developing a bespoke architecture, we adopt an Extreme Gradient Boosting regressor (XGBoost) [12], tuned via K-fold cross-validation, to map compact statistical descriptors of the event stream to the scintillometer-measured $C_{n}^{2}$. In doing so we: (i) compile and analyze a long-range, real-world dataset of synchronized event-camera and scintillometer measurements (which, to the best of our knowledge based on the literature surveyed, is among the earliest such field datasets reported), (ii) systematically quantify the impact of integration time and scene contrast on event-based turbulence sensing, (iii) demonstrate that an interpretable, modest-sized XGBoost model can achieve a Pearson correlation of ≈0.9 with ground truth while remaining computationally lightweight, and (iv) extract practical guidelines for sensor placement, target design and data-window selection that maximize estimation accuracy.

The remainder of this article is organized as follows. Section 2 reviews related work, covering deterministic turbulence sensors, passive imaging and machine learning approaches, and recent advances with neuromorphic event cameras. Section 3 details the experimental setup, including the 300 m test range, target-contrast configurations, event-camera and scintillometer instrumentation, data-collection schedule, and integration-time settings. Section 4 describes the methodology, outlining event-stream feature extraction, the three regression models (variance-based, event-count, and XGBoost), and the training/validation protocol. Section 5 presents the quantitative results, comparing model accuracies and analyzing the influences of exposure time and target contrast. Section 6 provides a discussion of key findings, practical advantages, limitations, and operational considerations. Section 7 concludes with the main contributions and directions for future research, including prospects for kilometer-scale paths and deep event-stream learning.

## 2. Related Work

### 2.1. Traditional Turbulence Estimation Methods

The refractive index structure parameter $C_{n}^{2}$ quantifies the strength of small-scale refractive-index fluctuations in the atmosphere. Under Kolmogorov turbulence theory, the second-order structure function of the refractive index is defined as:

(1) $$D_{n}\left(r\right)=\;\langle {\left[\left(x+r\right)-n\left(x\right)\right]}^{2}\rangle =C_{n}^{2}r^{2/3}\;,\;$$

which describes how refractive-index differences grow with spatial separation r.

A closely related quantity is the Fried coherence diameter $r_{0}$ widely used in imaging, adaptive optics, and free-space optical communication. For optical wavelength λ, it is given by:

(2) $$r_{0}=\;{\left[0.423k^{2}\int_{0}^{L}C_{n}^{2}\left(z\right)dz\right]}^{-3/5},\;$$

where k=2π/λ. The Fried parameter represents the transverse spatial scale over which the wavefront remains coherent and provides an equivalent measure of turbulence strength.

Typical atmospheric turbulence values along horizontal near-surface paths span multiple orders of magnitude. Under daytime convective conditions, $C_{n}^{2}$ commonly lies in the range $10^{-14}–10^{-12}\;m^{-2/3}$, while stable night-time conditions often fall below $10^{-15}\;m^{-2/3}$ [13]. Modern large-aperture scintillometers achieve absolute accuracies on the order of 10–20 percent, with documented cross-instrument biases of 5–15 percent in field intercomparison studies [14,15]. Because relative error scales inversely with the magnitude of $C_{n}^{2}$, percentage deviations can exceed 50–70 percent when the true turbulence level is very low, even when the absolute differences are small. This context helps interpret the error magnitudes discussed in Section 5.2 and Section 5.3.

The path-integrated refractive-index structure parameter $C_{n}^{2}$ remains the canonical metric for quantifying optical turbulence along ground and slant paths. Large-aperture scintillometers (LAS) dominate operational practice because they directly sense irradiance scintillation produced by turbulence-induced log-amplitude fluctuations [15,16]. Modern LAS designs use apertures of 10–15 cm to average inner-scale effects and achieve centimeter-level sensitivity over kilometer-length baselines. Multi-aperture and dual-wavelength variants further separate temperature and humidity contributions, while side-by-side inter-comparisons reveal relative biases on the order of 5–15% that must be removed by field calibration [15]. Angle-of-arrival (AoA) variance methods replace the active transmitter with a high-resolution imager focused on a point-like beacon or a distant edge; the random centroid jitter is proportional to the integrated phase gradient variance [17]. AoA techniques are fully passive, yet their dynamic range collapses when centroid motions approach sub-pixel scales under weak turbulence, and they are sensitive to platform vibration and tracking errors.

For profile-resolved sensing, wavefront-sensor arrays—Shack–Hartmann, curvature sensors, or recently pyramid sensors—recover spatial phase gradients across a pupil and, via a Fourier inversion, provide $C_{n}^{2}$ profiles with sub-kilometer resolution [18]. Combined with laser guide stars, these systems underpin modern astronomical adaptive optics and directed-energy test ranges; however, the requirement for a bright cooperative beacon, calibrated optics and high-bandwidth deformable-mirror control makes them impractical for lightweight, field-deployable monitoring. Dual-diode-laser Hartmann turbulence sensors can probe horizontal paths of up to 5 km and jointly retrieve $r_{0}$, the Greenwood frequency and inner-scale parameters, but still demand a free-beam link and careful co-alignment [19].

Recent reviews of optical coherence and turbulence sensing further expand this landscape. A comprehensive survey of atmospheric coherence length measurement techniques [20] summarizes both indirect approaches that retrieve the Fried parameter *r*_0_ from the refractive index structure parameter $C_{n}^{2}$ and direct coherence-length measurements, providing useful context for scintillation and wavefront-based sensing. In addition, Shack–Hartmann wavefront sensors have been used to estimate atmospheric coherence length from measured phase fluctuations along the propagation path. The work of Li et al. [21] derives an analytical expression for coherence length based on a Bump phase spectrum and validates it experimentally by collecting phase data with a Shack–Hartmann sensor, linking wavefront phase statistics to coherence length, which is directly related to the path-integrated $C_{n}^{2}$.

When optical hardware cannot be deployed, meteorological proxy models convert temperature variance, wind shear and humidity into surface-layer $C_{n}^{2}$ using Monin–Obukhov similarity theory. Basu showed that combining sonic anemometer data with similarity functions yields reasonable near-surface profiles [22], while feed-forward neural networks trained on tower measurements improve site-specific accuracy by learning local coefficients [23]. These methods offer useful boundary-layer estimates and short-term forecasts but lack the fidelity required for electro-optical system performance prediction without optical validation [3].

### 2.2. Passive Imaging and Data-Driven Methods

Passive, single-ended video-based estimation seeks to remove the transmitter entirely. Porat & Shapira pioneered the approach by measuring the variance of AoA fluctuations of a high-contrast checkerboard recorded at 30 fps; correlation coefficients of 0.8–0.9 with a co-located LAS were reported for moderate daytime turbulence [24]. Zamek & Yitzhaky generalized the technique by analyzing spatio-temporal image-gradient statistics in arbitrary natural scenes, demonstrating useful prediction accuracy when exposure times were kept below the coherence time of the turbulence [7]. Bose-Pillai et al. introduced differential feature-motion analysis of time-lapse imagery to retrieve $C_{n}^{2}$ and Fried’s coherence diameter, achieving <10% bias over a 1 km coastal path when compared with an LAS and scintillometer [5].

Frame-based passive methods, however, are fundamentally limited by the Nyquist barrier of conventional CMOS sensors: microsecond-scale scintillation is undersampled, and exposure times must trade motion blur against photon shot noise. To overcome these constraints, researchers have turned to machine learning (ML) models that learn non-linear turbulence signatures. Early statistical learners combined handcrafted features—gradient variance, temporal spectrum slopes, frame-difference histograms—using support-vector regression to predict $C_{n}^{2}$ in coastal-marine trials [25]. Vorontsov et al. trained a deep convolutional network on more than 50,000 retro-reflected laser-spot images and obtained <10% mean-absolute error for kilometer-scale links under $C_{n}^{2}>5\times 10^{-14}\;m^{-2/3}$ conditions [18]. Yet generalization gaps emerged: networks trained on a single target geometry or illumination failed when the scene changed. Saha et al. addressed this by embedding physics-based operators—such as a differentiable gradient-variance layer into a convolutional neural network )CNN( backbone, retaining deep representational power while constraining the solution space to physically plausible mappings; cross-site testing showed >30% lower RMSE than purely data-driven baselines [6].

Parallel work has targeted image restoration under turbulence. Cycle-consistent adversarial networks (CycleGAN-T) [26,27] and spatial-temporal Transformer models (TMT-Net) [28] reconstruct turbulence-degraded imagery with peak signal-to-noise gains of 3–5 dB and simultaneously output coarse $C_{n}^{2}$ estimates, hinting at dual-use architectures that recover both clean imagery and turbulence metrics. Despite these advances, frame-based ML retains the inherent dynamic-range (≈60 dB) and frame-rate (≤1000 fps with global-shutter CMOS) limits that challenge sensing of weak night-time scintillation or rapid, strong daytime scintillation.

### 2.3. Event Cameras and Neuromorphic Vision for Turbulence Sensing

Event-based cameras depart radically from frame paradigms by encoding only logarithmic brightness changes, each stamped with microsecond timing and pixel address. The resulting stream is sparse—often <1% duty cycle, yet captures intensity fluctuations up to >10 kHz with negligible motion blur and >120 dB dynamic range [8]. These properties align almost perfectly with the requirements of turbulence sensing, where useful information lies in sub-millisecond, low-contrast scintillation of natural edges and point sources.

Polnau & Vorontsov provided the first field demonstration, mounting a Prophesee sensor behind a 15 cm telescope. Using this setup, they showed that there is a correlation between the spatial variability of events and the measured value of $C_{n}^{2}$ over a 7 km trajectory across $10^{-15}–10^{-13}\;m^{-2/3}$ turbulence levels [11]. Boehrer et al. addressed turbulence mitigation rather than measurement: by clustering coherent spatio-temporal event patterns they separated true object motion from stochastic refractive jitter, reconstructing de-warped video in scenes with moving vehicles while preserving point-spread-function detail unavailable to 300 fps CMOS cameras [10].

Beyond atmospheric optics, a rich body of high-speed vision validates event sensors’ resilience. Brandli et al. demonstrated edge tracking of projectiles moving at 20 m/s with 3 µs latency [29]; Gehrig et al. achieved real-time eye tracking under 150 Hz strobe illumination by fusing events with a lightweight Kalman filter [30]. Such successes in high dynamic range (HDR) and microsecond domains underscore the potential of event cameras to capture the subtle brightness perturbations induced by turbulent air columns. Nevertheless, quantitative studies remain scarce: open questions persist on how integration-window length, contrast polarity thresholds and event-representation choices (rate maps, time surfaces, and spike tensors) affect estimation bias and variance across weak-to-strong turbulence regimes—issues the present work addresses through a systematic 2-to-50 s integration-time sweep and multi-contrast target design.

## 3. Experimental Setup

### 3.1. Site and Instruments

The field experiment was conducted between 27 March and 3 April 2025 at the Yavne site. The test location is situated on the coastal plain of central Israel (31.90° N, 34.75° E) at an altitude of approximately 40 m above sea level. The instrument layout provided a direct 300 m line-of-sight between the imaging sensors and the target panel along a near-ground horizontal path at near sea level. During the measurement campaign, ambient coastal-plain conditions were typical for early spring: daytime temperatures of approximately 18–26 °C, nighttime minima near 12 °C, relative humidity values ranging from roughly 50–70%, and light-to-moderate surface winds generally in the range of 1–4 m s^−1^. These ambient conditions characterize the meteorological environment within which the scintillometer, event camera, and CMOS camera acquired data, and provide contextual information for interpreting the turbulence regimes encountered during the experiment.

**Figure 1:**
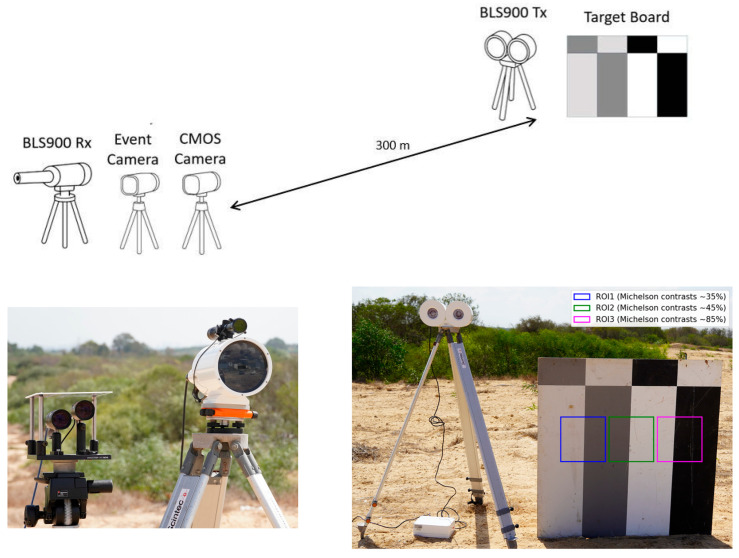
Experimental geometry and hardware. (**Top**): Schematic of the 300 m line-of-sight between the receiver station (BLS900 Rx co-aligned with the event and CMOS cameras) and the transmitter station (BLS900 Tx with the three-contrast target board). (**Bottom-left**): photograph of the receiver station; (**Bottom-right**): photograph of the transmitter and target; both terminals are mounted near ground level.

The event camera and CMOS camera were positioned side-by-side at the receiver station and were field-calibrated to share the same IFOV of 130 µrad. The CMOS camera (Basler acA2040-120um) captured high-speed video at 180 frames per second (fps), allowing it to record rapid turbulence-induced distortions. The event camera (Prophesee Metavision EVK-4 HD, with a Sony IMX636A sensor (Sony Semiconductor Solutions Corporation, Atsugi, Kanagawa, Japan)) asynchronously recorded changes in brightness as “events.” Both cameras were mounted close together and aligned with the scintillometer’s line-of-sight path, as illustrated in Figure 1.

### 3.2. Target and Contrast Levels

The imaging target was a vertical planar contrast panel placed near the scintillometer Tx. This panel featured three distinct vertical stripe patterns of different Michelson contrasts. The stripes provided low contrast (~35% Michelson, using a dark-gray vs. light-gray pair), medium contrast (~45%, dark-gray vs. white), and high contrast (~85%, black vs. white) regions. For completeness, the panel also included horizontal stripe patterns of similar contrasts, but these horizontal features were not utilized in the present study.

For analysis, regions of interest (ROIs) were defined around each vertical stripe pattern. Each ROI covered a 20 × 20 pixel area encompassing one set of vertical bars at the three contrast levels. We label these regions as ROI1 (low-contrast stripe), ROI2 (medium-contrast stripe), and ROI3 (high-contrast stripe). These ROIs provided localized image/event data corresponding to the different contrast conditions, enabling comparative evaluation of turbulence effects across low, medium, and high contrast imagery.

### 3.3. Sensors Configuration

Scintillometer: A Scintec BLS900 large-aperture scintillometer was deployed to provide reference measurements of atmospheric turbulence strength. The BLS900’s transmitter and receiver units were aligned along the 300 m path with optimal geometry. This instrument measures path-integrated refractive index fluctuations via optical scintillation and reports the corresponding structure parameter $C_{n}^{2}$ (refractive index structure constant) for the path [31]. For our experiment, the BLS900 was configured with a 10 s integration time, outputting a new $C_{n}^{2}$ value every 10 s. These $C_{n}^{2}$ readings (in $m^{-2/3}$) serve as the ground-truth turbulence strength against which the cameras’ observations were compared.

CMOS Camera: The frame-based camera was a Basler acA2040-120um HD CMOS video camera. This camera provides a spatial resolution of 2048 × 2048 pixels, a 5.5 µm pixel pitch, and a global-shutter architecture. Its spectral responsivity extends from the visible into the near-infrared (approximately 400–1000 nm), which is typical for industrial CMOS sensors. Although the sensor is sensitive up to ~1 µm, the illumination and target reflectance in our experiment were dominated by visible wavelengths, and the system effectively operated within the visible band.

It was operated in a high-speed mode (global shutter) at 180 fps to freeze turbulence-induced image distortions. To manage data volume, the CMOS camera was set to record synchronized 50 s video clips each minute (i.e., 50 s on, 10 s off), yielding effectively one 50 s capture per minute. This timing was chosen to align with the scintillometer’s 10 s sampling and to ensure overlapping data segments.

Event Camera: The neuromorphic event camera was a Prophesee Metavision EVK-4 HD, incorporating the Sony IMX636A event-based sensor. The IMX636A provides a spatial resolution of 1280 × 720 pixels with a 4.86 µm pixel pitch, a dynamic range exceeding 120 dB, and a spectral responsivity spanning approximately 400–1000 nm. This makes it suitable for capturing rapid brightness fluctuations across both visible and near-infrared wavelengths. The event sensor outputs asynchronous polarity events with microsecond latency based on brightness-change thresholds. For this experiment, we set its contrast sensitivity thresholds to *diff_on* = −36 and *diff_off* = −7 (dimensionless units). These thresholds were not hardware defaults; prior to the main campaign we conducted a short preliminary measurement at the same site to characterize event-rate behavior under representative daytime turbulence. Based on these trials, the thresholds were tuned so that, during peak afternoon turbulence, the entire scene generated approximately one million events per second. This configuration ensured sufficient event activity for robust feature extraction while preventing sensor saturation. No additional optical filters were used on the event camera.

The event camera was co-located with the CMOS camera, and both instruments were carefully boresighted to share the same instantaneous field of view (IFOV) of 130 µrad, ensuring that each ROI in the frame camera corresponded to the same physical region in the event data.

### 3.4. Data Collection

All sensors operated continuously 24/7 throughout the one-week experimental campaign. Data recording began on 27 March 2025 at 12:55 and ended on 3 April 2025 at 14:10 (local time). The scintillometer logged a $C_{n}^{2}$ measurement every 10 s during this entire period. The CMOS camera produced a 50 s high-speed video each minute (as described above), and the event camera simultaneously recorded a continuous stream of events. All devices, including the scintillometer, CMOS camera, and event camera were configured to use the same absolute system clock. This ensured that timestamps across all sensors were directly comparable, allowing each 10-s scintillometer interval to be precisely matched to the corresponding segment of the event stream and to overlapping CMOS video frames without temporal offset or interpolation. For the purposes of analysis, we focus on the daylight hours (07:00–19:00 local time each day) when the target was clearly visible. During these daytime periods, conditions were seasonally warm at Yavne, with air temperatures ranging from 24 to 34 °C, providing a broad span of convective turbulence strengths over the week. This yielded roughly 80 hours of usable multimodal recordings under varying turbulence conditions. All nighttime data (when the target contrast was insufficient for reliable event or image analysis) were set aside. The synchronized dataset from the three sensors (scintillometer, CMOS, and event camera) provides a rich record of turbulence effects over a range of conditions during the experiment.

### 3.5. Integration and Exposure Times

To facilitate direct comparison with the scintillometer and to investigate temporal smoothing effects, the raw event data were aggregated into fixed integration windows of varying durations. In post-processing, we accumulated the asynchronous events into equivalent frame-like counts or statistics over 5 distinct integration times: 2 s, 5 s, 10 s, 30 s, and 50 s. Each such window produces a summary of the event activity (e.g., an event count image or other statistical features as described in Section 4.1) corresponding to that time span. These specific durations were chosen to match or bracket the scintillometer’s 10 s reporting interval and to explore the trade-off between temporal resolution and measurement stability. Short integration windows (2–5 s) preserve higher temporal resolution, capturing rapid fluctuations in turbulence strength at the cost of increased variance in the estimates. In contrast, longer windows (30–50 s) provide more averaged, stable indicators of turbulence (reducing random noise or transient fluctuations) but at the cost of temporal responsiveness. By comparing results across different integration times, we can assess how the estimation of turbulence strength from event-based features is affected by the averaging duration, and identify an optimal balance between responsiveness and reliability for this application.

## 4. Methodology

### 4.1. Feature Extraction from Event Data

For each integration window (2–50 s) and each ROI, we computed a compact set of nineteen statistical features designed to capture spatial, temporal, and polarity characteristics of the event stream. Spatial features quantify horizontal and vertical wander (maximum span, mean span, standard deviation), two-dimensional dispersion, spatial entropy, and density. Temporal features describe event-rate behavior and inter-event timing (mean, standard deviation, and median of temporal gaps). Polarity features reflect the balance between positive and negative brightness changes. Together, these descriptors provide an expressive yet lightweight characterization of scintillation dynamics across the full turbulence range.

A concise summary of the feature set appears in Table 1, and the full extended table with formulas and definitions is provided in Appendix A. In general, increasing turbulence produces larger spatial spreads, higher event counts and entropy, shorter inter-event intervals, and stronger correlations between position and time. In weak turbulence, the opposite occurs: spreads contract, activity decreases, entropy falls, and temporal gaps widen.

These systematic trends allow the regression models in Section 4.2 to track $C_{n}^{2}$ reliably across the $10^{-14}$–$10^{-12}$ $m^{-2/3}$ range.

### 4.2. Proposed Prediction Models

Having extracted comprehensive statistical features, we developed three predictive models to estimate the turbulence strength ($C_{n}^{2}$) from event data. The first two are interpretable linear regressions based on single features for clarity, and the third is a multivariate ensemble model (XGBoost) leveraging all features.

#### 4.2.1. Linear Regression on Spatial Variance

**Figure 2:**
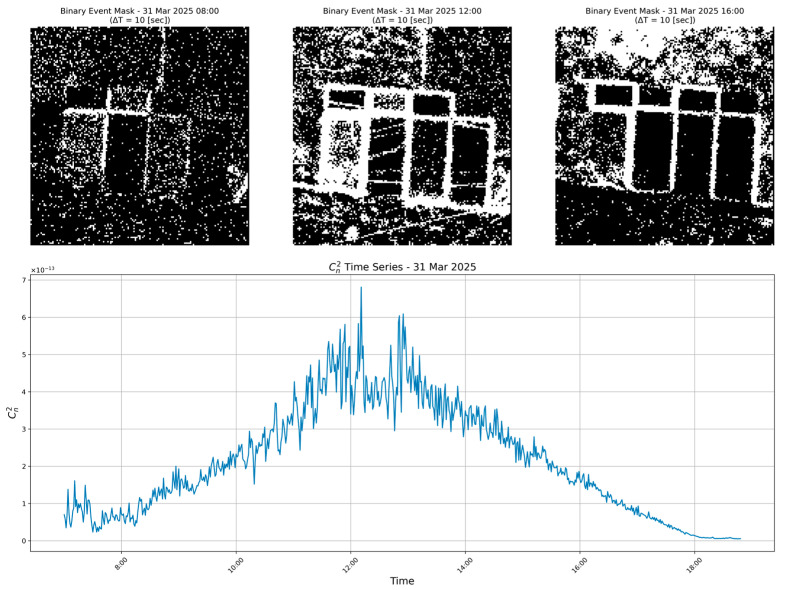
Binary event-accumulation masks (white = at least one event, black = none) for three 10 s windows—8:00 (weak), 12:00 (strong), 16:00 (moderate)—illustrate how turbulence broadens the active band. The lower panel plots the independent ground-truth $C_{n}^{2}$ time series measured by the BLS900 scintillometer (not inferred from events) for the same day, highlighting the midday peak that corresponds to the broadest masks.

Our first model uses a single feature—the standard deviation of vertical event positions (denoted STD Spatial Spread Y, see line 9 in Table 1)—as the predictor for turbulence strength. We chose this feature based on prior correlation-based studies [11]. In particular, Polnau and Vorontsov (2021) [11] observed that the spread of events along a building edge strongly correlates with $C_{n}^{2}$. Here, we extend that insight by training a linear regression to directly predict the $C_{n}^{2}$ value from the vertical spread (For examples of the dispersion as a function of different turbulence values, see Figure 2). The model is of the form ${C_{n}}^{2}={a\times STD}_{y}+b$ Fitted on our data. We employed a Mean Squared Logarithmic Error (MSLE) loss [32] for training (discussed in Section 4.3), which effectively means the regression minimizes the squared error in ${log}_{10}(C_{n}^{2}$). This choice ensures the model is sensitive to fractional errors equally across the wide range of turbulence values ($10^{-14}–\;10^{-12}\;m^{-2/3}$). By using MSLE, an under-prediction of a high $C_{n}^{2}$ and an over-prediction of a low $C_{n}^{2}$ are penalized more evenly [32,33].

Using only the STD Y feature, the linear model tracks turbulence reasonably in the strongest range (Range 5, $C_{n}^{2}\geq 4\times 10^{-13}\;m^{-2/3}$, where image wander dominates the signal. Outside that regime, however, a single spread metric cannot fully capture the variability: errors climb noticeably in moderate (Ranges 3 and 4) and weak (Ranges 1 and 2) conditions, where spatial jitter narrows and other factor, such as event-rate fluctuations, become more informative (Ranges 1–5 are defined in Section 4.4). In contrast, the “event-count” linear model (see Section 4.2.2) retains good accuracy through both strong and medium turbulence, highlighting that total activity better reflects mid-range scintillation than spread alone. Thus, while STD Y confirms the qualitative link between vertical images wander and $C_{n}^{2}$, its predictive power is confined mainly to high-turbulence windows; broader coverage requires additional or alternative features.

#### 4.2.2. Linear Regression on Event Count

**Figure 3:**
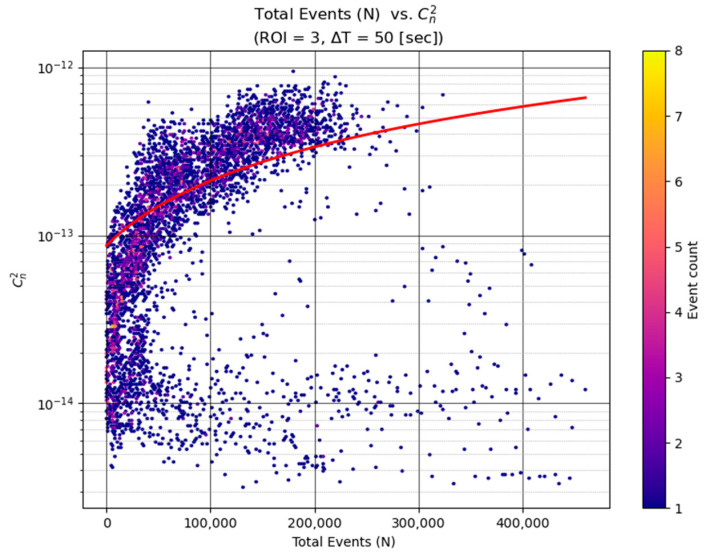
Log-linear heat map of total event count versus turbulence intensity $C_{n}^{2}$ for 50-s windows for a high-contrast region (ROI = 3). Each point represents a combination of event count versus measured value colored by density (yellow areas contain many overlapping samples). A clear positive correlation is evident—windows with more events tend to have higher $C_{n}^{2}$. The red line shows the fit of the linear regression model in space, illustrating the overall trend. That the event rate increases with turbulence intensity, especially from the mid to high range $C_{n}^{2}$.

Our second model uses another single predictor: the total number of events in the ROI window (see Figure 3). This linear regression was inspired by the observation that event rates tend to increase with turbulence. Intuitively, more intense turbulence causes more frequent brightness changes and hence more events. We found that especially in moderate to strong turbulence conditions, the event count correlates well with $C_{n}^{2}$—high turbulence levels produce a flurry of events, whereas low turbulence yields only a few events from occasional small waviness.

Similar to the first model, we fit a line ${C_{n}}^{2}=c\times (Total\;Events)+d$ using MSLE as the loss. The MSLE training ensured that both small counts (a few events in calm periods) and large counts (thousands of events in strong turbulence) were handled without biasing the fit toward one end of the range. That is, the model shows that in strong and moderate turbulence ranges there is an approximate linear relationship between the event count and $C_{n}^{2}$. In other words, the increase in the event count corresponds to a constant factor to the increase in the predicted $C_{n}^{2}$ value (albeit with some saturation at the edges).

Visualizes this relationship by plotting total event count against the corresponding scintillometer-measured $C_{n}^{2}$ for ROI 3 at ΔT=50 s, with a fitted regression curve overlaid. At very low turbulence levels, the event rate approaches the sensor’s noise floor, producing a cluster of low-activity points near the bottom of the plot. This behavior is expected in the weak-turbulence regime, where optical distortions fall below the event camera’s sensitivity. These points do not adversely affect the regression models because the learner correctly captures the nonlinear feature–response curve, and MSLE weighting prevents the low-turbulence samples from dominating the loss; furthermore, the associated absolute errors are inherently small in this regime.

Despite using only the scalar event count as input, this model achieves a reasonable predictive capability. In fact, for stronger turbulence (e.g., $C_{n}^{2}>\;10^{-13}\;m^{-2/3}$), the event count alone serves as a strong indicator of turbulence intensity. For very weak turbulence, the correlation is poorer—low counts can result from either truly quiescent air or just brief observation windows—but those conditions are less operationally critical. Like the previous model, this one was trained with MSLE to ensure predictive accuracy across the full dynamic range of $C_{n}^{2}$. The simplicity of this model makes it attractive for quick estimates: one could imagine a sensor simply counting events to gauge turbulence in real time. However, it ignores spatial information and other nuances, which motivates a more complex model.

#### 4.2.3. XGBoost Regression Model

The third and primary model is a multivariate regression that ingests all 19 features described in Section 4.1. We chose the XGBoost (Extreme Gradient Boosting) algorithm for this task, as it is well-suited for regression and can handle feature interactions and nonlinearities [12]. XGBoost is a gradient-boosted ensemble of decision trees, which can capture complex relationships between the event features and the turbulence strength.

**Hyperparameter tuning**: We performed extensive hyperparameter tuning to optimize the XGBoost model for each ROI and integration time. A random search of 3000 trials was conducted for each time window and ROI, out of about 324,000 possible unique hyperparameter combinations. All ROI/time configurations). The main XGBoost parameters optimized included:

- **max_depth**: the maximum tree depth (we searched values from shallow trees like 3 up to deep trees like 10+).
- **min_child_weight**: the minimum sum of instance weights needed in a child node (we varied this to control overfitting).
- **learning_rate**: the shrinkage rate for each boosting step (tested values from 0.01 to 0.3).
- **n_estimators**: the number of boosting rounds (ranging from 300 to 2000).
- **subsample**: the fraction of training samples used per tree (values from 0.6 to 1.0 to introduce randomness).
- **colsample_bytree**: the fraction of features sampled per tree (to prevent any single feature from dominating).
- **regularization parameters**: regularization parameters, including gamma, which specifies the minimum loss reduction required to create a new tree split, and lambda, which controls L2 weight regularization to prevent overfitting.

Each random combination was evaluated via cross-validation (details in Section 4.3), and the best-performing hyperparameters (in terms of validation MSLE loss) were selected. This extensive search ensured that the XGBoost model was neither underfit nor overfit, and that it made good use of the feature set. Notably, the optimal settings tended to use max_depth in the range of 7–9 (allowing moderately complex interactions), a learning_rate around 0.01–0.05 (slow learning for stability), and a large number of trees (hundreds to a thousand) indicating the benefit of gradual boosting. The final model, with all hyperparameters tuned, was then trained on the full training set and applied to the test folds for evaluation.

In summary, the XGBoost regression combines all the spatial, temporal, polarity, and statistical features to produce a turbulence strength estimate. We hypothesize that this model can learn subtle patterns—for instance, it might use a combination of high event count and high polarity alternation to recognize strong scintillation, or use a moderate spatial spread plus a high active pixel fraction to identify moderate turbulence versus merely a moving object. The exact learned feature importances will be analyzed later, but the methodology here is designed to let the data speak: by providing a rich feature set and using a powerful learner with thorough tuning, we aim to capture the complex mapping from event signatures to $C_{n}^{2}$.

### 4.3. Training and Validation

All models were trained and evaluated with three-fold cross-validation applied to the entire multi-day dataset. We concatenated all day-time windows (27 March–3 April 2025, 07:00–19:00) into one chronological sequence and then split that sequence into three equal, contiguous blocks. In each fold two blocks served as the training set and the remaining block as the test set, so every time segment was held out exactly once. This time-ordered, non-overlapping partition safeguards against temporal leakage and yields a realistic test of generalization across different days and diurnal turbulence cycles.

For the regression objective, we used the Mean Squared Logarithmic Error as the loss function during training for all models. As noted in Section 4.2.1 and Section 4.2.2, MSLE treats errors on a log scale, which is appropriate because $C_{n}^{2}$ values span several orders of magnitude. This choice is supported in the literature—studies in physical domains often prefer MSLE when the target can vary by decades (e.g., [34]). Saha et al. (2022) explicitly advocate for MSLE to balance prediction of small and large values in high-energy physics regression tasks [6], and we found the same principle applies here. By minimizing $\frac{1}{N}\sum_{i=1}^{N}{\left({log}_{e}\left(1+\;y_{i}\right)-\;{log}_{e}\left(1+\;\hat{y_{i}}\right)\right)}^{2}$, the models achieved balanced sensitivity: a $10^{-14}\;vs.\;2\times 10^{-14}$ error impacts the loss similarly to a $10^{-12}\;vs.\;2\times 10^{-12}$ error, percentage-wise. This is physically meaningful, since a small absolute error at a high $C_{n}^{2}$ (which might still be a large percentage error) should not be considered trivial. Prior works have recommended MSLE in such scenarios [35,36], and our results concur.

It is worth noting that the scintillometer outputs used for ground-truth $C_{n}^{2}$ are themselves 10-s averages. We synchronized each event-data integration window to exactly align with a scintillometer averaging interval, so that each training label corresponds to the same 10 s period of events. No interpolation was needed. During cross-validation splitting, we ensured that if a certain 10 s window was in the test set, the adjacent windows (before and after) were not used for training either (a small buffer)—this prevents any spatial leakage between ROIs due to the slight temporal correlation between consecutive $C_{n}^{2}$ measurements.

Model performance was evaluated on the held-out folds using error metrics described next. We also checked for any training vs. validation loss discrepancies to detect overfitting. In all cases, the linear models showed stable training (being very simple), and the XGBoost model, with the chosen hyperparameters and regularization, did not exhibit overfitting—the 3-fold average training loss was very close to the validation loss (difference within ~5%). This gives confidence that our models will generalize well to new days.

### 4.4. Error Metric

Model performance is assessed primarily with the Mean Absolute Relative Error (MARE), which offers a scale-independent view of percentage deviation. Let $y_{i}^{true}$ denote the ground truth scintillometer value for sample i, let $y_{i}^{pred}$ denote the model prediction, and let N be the total number of samples.

(3) $$MARE=\frac{1}{N}\sum_{i=1}^{N}\left|\frac{{y_{i}}^{true}-{y_{i}}^{pred}}{{y_{i}}^{true}}\right|\times 100\%,\;$$

where $y_{i}$ is the ground truth $C_{n}^{2}$ and $\hat{y_{i}}$ is the model estimate. In words, MARE is the average of the absolute errors normalized by the true value, expressed as a percentage. We chose MARE because it provides an interpretable percentage error and, importantly, weights errors in a scale-invariant way. An absolute error of $10^{-13}$ has very different importance if the true value was ${5\times 10}^{-13}$ (20% error) versus if the true value was ${5\times 10}^{-14}$ (200% error). Using MARE allows us to fairly evaluate performance across the full range of weak to strong turbulence by considering the error relative to the magnitude of $C_{n}^{2}$.

In addition to overall MARE, we performed a stratified error analysis by dividing the turbulence strength into five logarithmic ranges (levels of severity) and computing the error within each range. The ranges (in $C_{n}^{2}$ units of $m^{-2/3}$) were defined as:

- **Range 1**: $10^{-14}$ to ${3\times 10}^{-14}$ (very weak turbulence)
- **Range 2**: ${3\times 10}^{-14}$ to ${6\times 10}^{-14}$ (weak)
- **Range 3**: ${6\times 10}^{-14}$ to ${2\times 10}^{-13}$ (moderate)
- **Range 4**: ${2\times 10}^{-13}$ to ${4\times 10}^{-13}$ (strong)
- **Range 5**: ${4\times 10}^{-13}$ to $10^{-12}$ (very strong turbulence)

These bins each span roughly a factor of 2 in $C_{n}^{2}$ (except the last, which is a slightly larger factor). By evaluating, for example, the MARE within each bin, we can determine if a model performs better in certain turbulence regimes. This stratification is useful because a model might excel at detecting strong turbulence (small relative errors at high $C_{n}^{2}$) but struggle at very low turbulence (where predicting a small absolute value can lead to a high relative error), or vice versa. Indeed, in our results we will report the error in each range to highlight such behaviors. Overall, the use of MARE in combination with these turbulence bins provides a comprehensive and fair assessment of model accuracy across the spectrum of conditions from quiescent to highly turbulent.

Finally, in addition to the MARE, we compute the Pearson correlation coefficient ρ ∈ [−1, 1] between predicted and measured $C_{n}^{2}$. The terms ${\bar{y}}^{true}$ and ${\bar{y}}^{pred}$ denote the sample means of the true and predicted values, respectively.

(4) $$\rho =\frac{\sum_{i}\left({y_{i}}^{pred}-{\bar{y}}^{pred})({y_{i}}^{true}-{\bar{y}}^{true}\right)}{\sqrt{\sum_{i}{\left({y_{i}}^{pred}-{\bar{y}}^{pred}\right)}^{2}}\sqrt{\sum_{i}{\left({y_{i}}^{true}-{\bar{y}}^{true}\right)}^{2}}},\;$$

Pearson correlation captures trend fidelity, how well a model tracks day-to-day or minute-to-minute fluctuations, whereas MARE quantifies the average magnitude of the error. Together, these two metrics give a balanced view: a model may exhibit low relative error yet poor correlation if it predicts the mean well but misses temporal dynamics, or vice versa. Throughout Section 5 we report both MARE and ρ values to provide a comprehensive evaluation of each approach.

## 5. Results

### 5.1. Models Comparison

We developed and tested three models for predicting the turbulence strength ($C_{n}^{2}$) from event-based observations: (1) a variance model using the variance of image intensity in the region of interest (ROI) as a predictor, (2) an event count model using the number of events detected in the ROI over a given interval, and (3) an XGBoost model, which is a learning-based regression that leverages the event data to predict $C_{n}^{2}$. The models’ predictions were compared against ground-truth $C_{n}^{2}$ measurements, with accuracy evaluated using the Mean Absolute Error (MAE) and Mean Absolute Relative Error (MARE).

**Figure 4:**
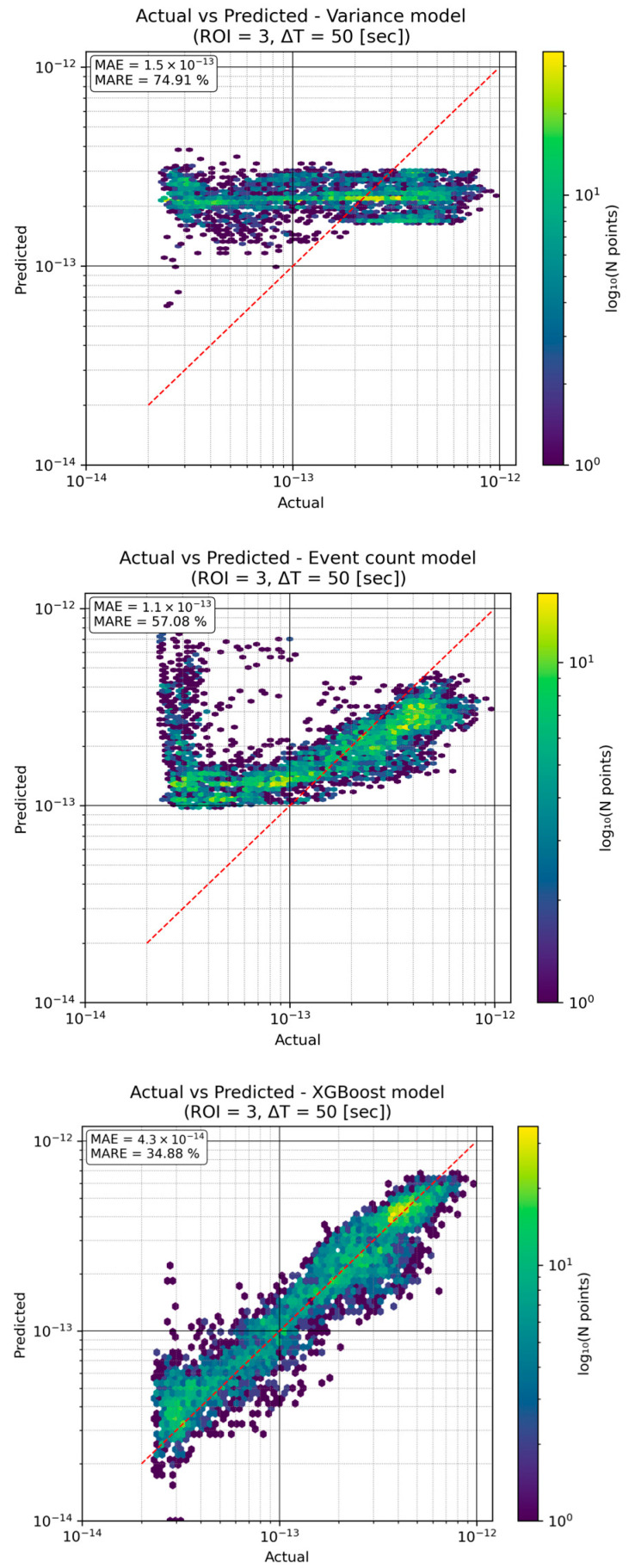
Actual vs. Predicted $C_{n}^{2}$ for the three models (ROI3, ∆T = 50 s). Each panel plots the model’s predicted $C_{n}^{2}$ against the true measured value; the red dashed line is the ideal y = x (perfect prediction) line. The variance model (**upper**) exhibits large scatter and a tendency to under-predict high $C_{n}^{2}$ values ($MAE\sim 1.5\times 10^{-13}$, MARE~75%). The event count model (**middle**) improves upon this ($MAE\sim 1.1\times 10^{-13}$, MARE~57%), but still shows substantial deviation from the ideal line. The XGBoost model (**lower**) achieves much higher accuracy, with points tightly clustered along the diagonal ($MAE\sim 4.3\times 10^{-14}$, MARE~35%). Similar patterns were observed for all contrast and integration-time combinations.

Alongside MAE and MARE, we also computed the Pearson correlation coefficient (ρ) between the actual and predicted values to quantify trend fidelity. For the high-contrast (ROI 3) with a 50 s integration window the variance model achieved ρ = 0.06, the event-count model achieved ρ = 0.48, and the XGBoost model reached ρ = 0.93. all three plots presented in Figure 4 confirm that only the learning-based model reliably follows the minute-to-minute fluctuations in turbulence strength, while the variance model captures virtually none of the temporal variation and the event-count model tracks it only moderately.

As seen in Figure 3, higher turbulence levels produce a greater number of events, which underpins the event count model’s approach. (By contrast, the intensity-variance feature showed a weaker correlation with $C_{n}^{2}$ in low-contrast conditions as presented in Section 5.3 and demonstrated in Figure 6, which likely limits the variance model’s performance.) Next, we examine the actual prediction performance of each model under the same representative conditions (using the high-contrast ROI = 3 and ∆T = 50 s integration time).

For this test scenario, the XGBoost model clearly outperforms the simpler models, achieving roughly half the relative error of the event-count model and only about one-third that of the variance model. The variance-based approach struggles especially at higher $C_{n}^{2}$, many points in Figure 4 (upper) fall well below the unity line, indicating under-estimation of strong turbulence. The event-count model tracks the overall trend better, but still with considerable scatter. In contrast, the XGBoost predictions lie much closer to the true values across the range.

Permutation importance reveals that five features together account for ≈45% of XGBoost’s explanatory power: (1) Total Events, (2) Inter-Event Std, (3) Positive Event Ratio, (4) Mean Spatial Span Y, and (5) Event Rate. The dominance of activity-level and timing cues explains why the event-count model already performs better than the variance model, while the added spatial-spread and polarity terms enable XGBoost to retain accuracy across weak, moderate, and strong turbulence. The prominence of metrics reflecting the vertical axis reflects the fact that only vertical bars were present in ROI 3.

In summary, a data-driven model (XGBoost) leveraging the full event feature set captures the complex, non-linear relationship between event statistics and $C_{n}^{2}$ far more effectively than either single-feature regression.

### 5.2. Integration Results

We next evaluated how the integration time ∆T (the duration over which events are aggregated for each data point) influences prediction accuracy. Intuitively, a longer integration window yields more event data and should improve the estimate, especially when the turbulence-induced signal is weak. However, longer ∆T also means slower update rates, so it is important to understand the trade-off.

**Figure 5:**
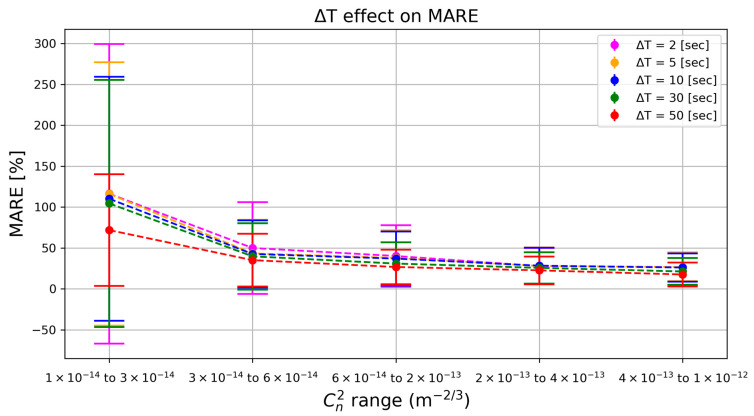
Mean Absolute Relative Error (MARE) achieved by the XGBoost model for five integration times (2 s, 5 s, 10 s, 30 s, 50 s) across the five ${CN}^{2}$ ranges. Shorter windows incur large errors at very weak turbulence (≈100% MARE for ΔT = 2 s in Range 1); extending the window to 50 s cuts that error to about 70%. For moderate and strong turbulence (Ranges 2–5) the curves converge, with all ΔT values dropping to MARE in the tens of percent or lower.

As shown in Figure 5, using a very short integration time (e.g., ∆T = 2 s) leads to high relative error when turbulence is weak, the model has very little data (few events) to go on, resulting in noisy estimates. Increasing ∆T provides more event observations and dramatically improves accuracy in those low-$C_{n}^{2}$ conditions (for example, MARE drops from about 100% at 2 s to around 70% at 50 s for the weakest turbulence bin). In stronger turbulence conditions (moving to higher $C_{n}^{2}$ ranges), even short windows capture plenty of events, so the benefit of longer integration diminishes and the error rates for all ∆T converge to similarly low values.

Pearson correlation coefficients tell the same story from a trend-tracking viewpoint: ρ climbs steadily from 0.84 (ΔT = 2 s) to 0.85 (5 s), 0.86 (10 s), 0.89 (30 s), and reaches 0.93 at 50 s. The incremental gains are modest above 10 s but show that longer integrations suppress noise and let the model follow minute-to-minute fluctuations more faithfully, especially in the weak-turbulence regime.

In summary, longer integration windows markedly improve prediction reliability in very weak turbulence by increasing event counts, while imposing only minor accuracy gains once turbulence is moderate or strong. Pearson ρ values confirm that trend fidelity follows the same upward trajectory, plateauing as soon as the window is long enough to stabilize the statistics.

### 5.3. Contrast Analysis

Finally, we analyzed the impact of scene contrast on the estimation accuracy. Different regions of interest were tested corresponding to varying inherent contrast in the imagery: ROI1 (low-contrast region), ROI2 (medium contrast), and ROI3 (high contrast). For this analysis, the integration time was fixed at ∆T = 50 s to isolate the effect of contrast.

**Figure 6:**
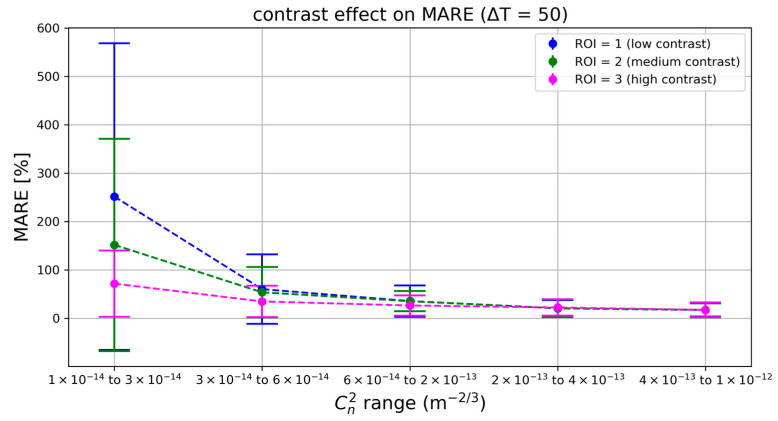
Effect of scene contrast on prediction error (MARE vs. $C_{n}^{2}$) achieved by the XGBoost model at a fixed integration time of 50 s. Results are shown for a low-contrast ROI (ROI = 1), medium-contrast ROI (ROI = 2), and high-contrast ROI (ROI = 3). In weak turbulence conditions (leftmost $C_{n}^{2}$ bin), the high-contrast region yields much lower error (~100% MARE) compared to the low-contrast region (MARE exceeding 200%). At higher turbulence levels, all three curves decline and eventually converge to low MARE values (a few tens of percent or less in the rightmost bin), indicating that when turbulence is strong, even a low-contrast region produces sufficient event data for a decent prediction.

Figure 6 highlights that scene contrast is a critical factor when turbulence-induced distortions are small. In the lowest $C_{n}^{2}$ range, the model’s error is enormous for the low-contrast ROI—the event signal is so faint that the $C_{n}^{2}$ estimates become unreliable (relative errors well over 200%). The medium contrast ROI performs better, and the high-contrast ROI performs best, with roughly half the error of ROI = 1 in that weak-turbulence regime. This gap shrinks as $C_{n}^{2}$ increases: under more intense turbulence, even a low-contrast scene yields plenty of events, allowing the model to achieve reasonably good accuracy. By the highest turbulence bin, all three ROI curves reach similar low error levels.

Pearson correlation corroborates these findings: with ΔT = 50 s, ρ = 0.918 for ROI 1, ρ = 0.917 for ROI 2, and ρ = 0.930 for ROI 3. Although the global correlations differ only slightly, the range-wise analysis reveals that the high-contrast ROI maintains stronger local correlations (and lower variance) in the weakest turbulence bin, whereas all three ROIs attain similarly high ρ values once turbulence exceeds 3 ×${\;10}^{-14}\;m^{-2/3}$.

In summary, using a high-contrast observation region significantly improves $C_{n}^{2}$ estimation accuracy in gentle turbulence conditions, whereas under strong turbulence conditions contrast becomes less critical since the signal is naturally stronger.

### 5.4. Conclusions

In conclusion, the results of this chapter underscore several key points regarding event-based turbulence measurement:

- **Model selection**: The learning-based XGBoost model dramatically outperformed the simpler variance- and event count-based models, achieving much lower error (MARE ~ 35% vs. 57–75% for the others under the test scenario). A more sophisticated model is clearly beneficial for capturing the nonlinear relationship between event data and $C_{n}^{2}$.
- **Integration time**: Longer integration times greatly improve accuracy when the turbulence signal is weak. For instance, extending ∆T from 2 s to 50 s reduced the error by roughly half in the lowest $C_{n}^{2}$ range. However, beyond a certain point, increasing ∆T yields diminishing returns, especially once turbulence is strong enough that even short samples contain ample event data.
- **Image contrast**: High-contrast imagery substantially lowers the prediction error in low-turbulence conditions. In a low-contrast region, the model’s error was 2–3 times higher than in a high-contrast region at the weakest turbulence levels. Under high turbulence, this contrast advantage fades as all regions produce sufficient event activity for accurate predictions.

Overall, to maximize the accuracy of $C_{n}^{2}$ estimates using an event-based sensor, one should use a robust model (like XGBoost), ensure a sufficiently long integration time for the given conditions, and prefer high-contrast observation regions. By optimizing these factors, our approach can reliably recover atmospheric turbulence strength across a range of conditions, with the greatest gains seen in challenging low-turbulence scenarios.

## 6. Discussion

The present campaign confirms that an event-based vision sensor can serve as a compact scintillometer surrogate, delivering real-time line-averaged $C_{n}^{2}$ with sub-minute latency. Across 80 hours of daylight data the XGBoost regressor achieved a global Pearson coefficient of 0.93 and a MARE of 35%, halving the error of the single-feature event-count model and cutting the variance model’s error by two-thirds (Figure 4). Trend fidelity remained high even on days when $C_{n}^{2}$ spanned two decades, from the pre-dawn floor $10^{-14}\;m^{-2/3}$ up to a noon peak of $10^{-12}\;m^{-2/3}$, underscoring the sensor’s ability to track rapid convective changes. Feature-importance analysis revealed that Total Events, Inter-Event Std, Positive Event Ratio, Mean Span Y and Event Rate explain 45% of the model variance. These dominant descriptors align with earlier field work: Polnau and Vorontsov reported a tight correlation between event spread and scintillometer readings over a 7 km path [11], while Boehrer et al. showed that temporal clustering of events carries turbulence information independent of spatial jitter [10].

Three practical advantages emerge. First, the sensor’s microsecond latency and >120 dB dynamic range capture high-frequency scintillation that frame cameras attenuate or blur [8]. Second, because only brightness changes are transmitted, the data rate scales with scene dynamics rather than pixel count, yielding bandwidths two orders of magnitude lower than 180 fps CMOS video for the same path. Third, the asynchronous output inherently suppresses static clutter, simplifying downstream processing and enabling facile motion–turbulence separation, a property leveraged by Boehrer et al. to stabilize backgrounds while preserving moving vehicles [10].

Parameter sweeps highlight two points. Increasing the integration window (i.e., ∆T) from 2 s to 50 s had little effect above $C_{n}^{2}=3\times 10^{-14}\;m^{-2/3}$ but cut weak-range (range 1) error from ∼100% to 70%. This suggests that adaptive windowing, short in strong seeing, long in calm air—could equalize accuracy without sacrificing responsiveness. Similarly, target contrast proved critical only in the lowest bin: at $C_{n}^{2}=3\times 10^{-14}\;m^{-2/3}$ the high-contrast ROI outperformed the low-contrast ROI by a factor of two in MARE, whereas in stronger ranges the curves converged. An obvious remedy for passive scenes is to raise the sensor’s ON/OFF thresholds when event rates soar and lower them when the stream becomes sparse; frequency-adaptive bias tuning for event cameras has already been demonstrated in hardware [37,38].

The study nonetheless has limitations. The 1280 × 720 sensor limits spatial resolution, and our target fills only ~60 × 60 px, so fine wavefront structure is averaged. This phenomenon can also explain the relatively poor performance of the regression model based on spatial variation compared to event count data. The experiment used a single 300 m path; kilometer-scale links or vertical slants could introduce inner-scale effects not captured here. Very weak turbulence still yields few events: even a 50 s window left Range 1 errors at ~70%, indicating that either longer dwell times or higher electronic gain will be needed for precision micro-seeing. Finally, the method depends on visible high-contrast structure; scenes such as a clear sky or uniform desert would require natural edges or artificial beacons.

Although this study focuses on a 300 m horizontal path, the methodology is inherently transferable to other geometries. The event-based features respond to scintillation statistics associated with the path-integrated $C_{n}^{2}$, so the same sensing principle applies to longer baselines provided the target occupies a similar angular footprint and the turbulence range remains comparable. Very long paths may require retraining to account for stronger log-amplitude fluctuations or saturation. Vertical propagation is also compatible with our framework: the feature set captures spatial dynamics in both axes and temporal variability, making it geometry-agnostic. A model trained on vertical-path data should therefore learn the appropriate mapping for stratified atmospheric conditions.

Beyond propagation geometry, the method is not limited to the specific stripe target used in this campaign. The features we extract, like spatial jitter, event-rate statistics, polarity ratios, and temporal dynamics, are agnostic to target identity and only require sufficient edge contrast to generate a stable event stream. Our contrast analysis (Section 5.3) indicates that even modest-contrast regions can support estimation in moderate and strong turbulence. Straight edges, corners, or natural scene features should therefore be usable with appropriate retraining. Evaluating performance on purely natural targets is an important direction for future work.

Although this study was conducted at a single site in Yavne, Israel, the underlying sensing principle is environment agnostic. The event-based features we use, such as spatial jitter, temporal variability, polarity ratios, and entropy, are direct signatures of optical scintillation and are therefore applicable across different terrains, elevations, and weather regimes. What changes between locations is the distribution of $C_{n}^{2}$ and the dominant convection mechanisms. The methodology can thus be applied in other environments, although the regression model would benefit from retraining on representative local data to capture site specific characteristics. Evaluating cross site generalization is an important direction for future work.

Frame-based estimators, e.g., the gradient-variance method of Zamek & Yitzhaky [7], remain an important benchmark. We collected 180 fps CMOS video during the same campaign and will report a head-to-head comparison separately. We expect the CMOS approach to excel in spatial resolution but to be less responsive to sub-frame turbulence bursts, mirroring findings from earlier two-camera studies [39].

## 7. Conclusions

This paper introduces a fast, single-ended technique for estimating atmospheric turbulence strength with a neuromorphic event sensor. By extracting nineteen lightweight features and training an XGBoost regressor, we achieved sub-40% relative error across $10^{-14}–10^{-12}\;m^{-2/3}$ without external illumination or moving parts. The work demonstrates that event cameras, long valued for robotics and high dynamic range vision, also furnish quantitative path-integrated turbulence data, opening a path toward portable scintillometers and real-time adaptive-optics aids [8,11].

The results imply that event-based sensing could substantially improve both the monitoring of atmospheric turbulence (via real-time, high-refresh-rate metrics) and imaging through turbulence (by enabling mitigation strategies that leverage sparse, high-temporal-resolution data) [8,10,11]. One key implication is that systems requiring optical propagation—long-range surveillance, free-space optical communications, targeting and navigation—can embed an event sensor alongside the main aperture to report live seeing and cue adaptive correction while maintaining low data rates and resistance to motion blur [8,10].

Future work will extend the dataset and apply deep neural networks, which have shown strong performance for turbulence restoration and related inverse problems (e.g., GAN-based video de-turbulence) but require larger, diverse corpora [39]. A particularly promising direction is joint inference with a conventional CMOS camera: recent work demonstrates precise spatial synchronization and fusion of event/CMOS streams using CNN–DGCNN (Dynamic Graph CNN) models, suggesting that complementary spatial detail (frames) and temporal spikes (events) can be combined for superior estimation [8,40,41]. Beyond synchronization, such hybrid fusion has strong potential to improve $C_{n}^{2}$ estimation itself, especially in weak or low-contrast regimes where the two sensing modalities offer complementary information. Long-distance trials (kilometer-scale) and comparisons against established instruments will test range scalability and absolute accuracy, building on prior field demonstrations of event-based sensing and on classical frame-based estimators that serve as baselines [7,11]. We also aim to eliminate dedicated targets by tracking natural edges for truly passive operation, leveraging event-based robustness to separate turbulence from genuine object motion in outdoor scenes [10]. Finally, closed-loop bias control—adapting ON/OFF thresholds to ambient dynamics—offers a principled route to keep event density near an optimal operating point across weak and strong seeing, as suggested by recent frequency-adaptive tuning strategies for event cameras [37,38]. Collectively, these steps will advance event-based turbulence sensing from proof-of-concept to a robust field instrument for atmospheric optics.

## Figures and Tables

**Table 1:** Event stream features, formulas, and turbulence rationale.

#	Feature (Name)	Formula	Category	Intuition for Turbulence Estimation
1	Total Events	$N_{evt}$	Temporal	Event activity increases with scintillation.
2	Positive Event Ratio	$\frac{N_{+}}{N_{evt}}$	Polarity	Polarity bias in contrast changes.
3	Negative Event Ratio	$\frac{N_{-}}{N_{evt}}$	Polarity	Complement of above.
4	Max Spatial Span X	$max\left(x\right)-min(x)$	Spatial	Horizontal wander range.
5	Max Spatial Span Y	$max\left(y\right)-min(y)$	Spatial	Vertical wander; correlates with ${CN}^{2}$
6	Mean Spatial Span X	$\frac{1}{N_{evt}}\sum \left(x-min(x\right)$)	Spatial	Average horizontal displacement
7	Mean Spatial Span Y	$\frac{1}{N_{evt}}\sum \left(y-min(y\right)$)	Spatial	Average vertical displacement
8	STD Spatial Span X	$\sigma_{x}$	Spatial	Jitter magnitude (horizontal).
9	STD Spatial Span Y	$\sigma_{y}$	Spatial	Jitter magnitude (vertical).
10	Event Rate (MEvt/s)	$\frac{N_{evt}}{10^{6}T}$	Temporal	Normalized activity level.
11	XY Correlation	$\rho_{xy}$	Spatial	Detects diagonal shear.
12	XT Correlation	$\rho_{xt}$	Temporal	Horizontal drift trend.
13	YT Correlation	$\rho_{yt}$	Temporal	Vertical drift trend.
14	Spatial Event Density	$\frac{N_{evt}}{Area}$	Spatial	Density of scintillation activity.
15	Spatial Entropy (32-bin)	32 bin entropy	Spatial	Uniform scatter → stronger turbulence
16	Inter-Event Mean	$\bar{∆t}$	Temporal	Faster fluctuations → smaller gaps.
17	Inter-Event STD	$\sigma_{∆t}$	Temporal	Timing variability.
18	Inter-Event Median	Median(∆t)	Temporal	Robust temporal spacing.
19	Spatial Dispersion	RMS of 2d jitter	Spatial	Overall wander magnitude.

## Data Availability

The data presented in this study are available on request from the corresponding author.

## Appendix A

### Appendix A.1. Detailed Feature Definitions and Formulas

This appendix provides the full expanded version of the feature table used for the regression models. While Table 1 in the main text presents a concise summary, the table below includes all formulas, detailed descriptions, and the physical rationale behind each feature.

**Table A1:** Full event-stream feature set: definitions, formulas, and turbulence rationale.

#	Feature (Name)	Formula	Category	Intuition for Turbulence Estimation
1	Total Events	$N_{evt}$	Temporal	More turbulence → more brightness changes → larger $N_{evt}$
2	Positive Event Ratio	$\frac{N_{+}}{N_{evt}}$	Polarity	Deviation from 0.5 indicates bias in intensity changes
3	Negative Event Ratio	$\frac{N_{-}}{N_{evt}}$	Polarity	Complement to (2)
4	Max Spatial Span X	$max\left(x\right)-min(x)$	Spatial	horizontal image wander range
5	Max Spatial Span Y	$max\left(y\right)-min(y)$	Spatial	Vertical wander range; strongly linked to ${CN}^{2}$ [11]
6	Mean Spatial Span X	$\frac{1}{N_{evt}}\sum \left(x-min(x\right)$)	Spatial	Average horizontal displacement after offset removal
7	Mean Spatial Span Y	$\frac{1}{N_{evt}}\sum \left(y-min(y\right)$)	Spatial	Average vertical displacement
8	STD Spatial Span X	$\sigma_{x}=\sqrt{\frac{1}{N_{evt}}\sum x-\min\left(x\right)-\mu_{x}}$	Spatial	Robust spread of x; large under strong turbulence.
9	STD Spatial Span Y	$\sigma_{y}=\sqrt{\frac{1}{N_{evt}}\sum y-\min\left(y\right)-\mu_{y}}$	Spatial	Robust spread of y; large under strong turbulence. Principal predictor in Section 4.2.1
10	Event Rate (MEvt/s)	$\frac{N_{evt}}{10^{6}T}$	Temporal	Normalizes count by window length T (T measured in µsec)
11	XY Correlation	$\rho_{xy}=\frac{cov(x,y)}{\sigma_{x}\sigma_{y}}$	Spatial	Detects diagonal shear in displacements
12	XT Correlation	$\rho_{xt}=\frac{cov(x,t)}{\sigma_{x}\sigma_{t}}$	Temporal	Horizontal drift trend vs. time
13	YT Correlation	$\rho_{yt}=\frac{cov(y,t)}{\sigma_{y}\sigma_{t}}$	Temporal	Vertical drift trend vs. time
14	Spatial Event Density	$\frac{N_{evt}}{\left(max\left(x\right)-min(x))(max\left(y\right)-min(y)\right)}$	Spatial	Distinguishes dense scintillation from sparse wander
15	Spatial Entropy (32-bin)	$-\sum_{i=1}^{32}p_{i}log(p_{i})$	Spatial	Uniform scatter (high entropy) ⇔ strong turbulence
16	Inter-Event Mean	$\bar{∆t}=\frac{1}{N_{evt}-1}\sum {∆t}_{i}$	Temporal	Shorter mean gap = faster fluctuations
17	Inter-Event STD	$\sigma_{∆t}=\sqrt{\frac{1}{N_{evt}-1}\sum {\left({∆t}_{i}-\bar{∆t}\right)}^{2}\;}$	Temporal	Variability of event timing
18	Inter-Event Median	$Median\;of\;{∆t}_{i}$	Temporal	Robust central gap measure
19	Spatial Dispersion	$\sqrt{{\sigma_{x}}^{2}+{\sigma_{y}}^{2}}$	Spatial	Net 2D jitter; analogous RMS spot size

**Variable definitions:** x,y : pixel coordinates of each event inside the ROI; t : event timestamps (µs); $N_{evt}$ : total number of events; $N_{+}N_{-}$ : count of positive/negative events; $\mu_{x},\mu_{y}$ : means of $x-\min\left(x\right)$ and y−min(y); ${∆t}_{i}$: consecutive inter-event intervals within the window; $p_{i}$: probability of mass in the $i^{th}$ spatial bin for entropy; T: integration-window duration.
